# Density‐dependent resource partitioning of temperate large herbivore populations under rewilding

**DOI:** 10.1002/eap.70090

**Published:** 2025-09-15

**Authors:** Eduard Mas‐Carrió, Perry Cornelissen, Han Olff, Luca Fumagalli

**Affiliations:** ^1^ Laboratory for Conservation Biology, Department of Ecology and Evolution, Biophore University of Lausanne Lausanne Switzerland; ^2^ State Forestry Service Amersfoort The Netherlands; ^3^ Institute for Biodiversity and Ecosystem Dynamics University of Amsterdam Amsterdam The Netherlands; ^4^ Conservation Ecology Group, Groningen Institute for Evolutionary Life Sciences University of Groningen Groningen The Netherlands; ^5^ Swiss Human Institute of Forensic Taphonomy, University Centre of Legal Medicine Lausanne‐Geneva Lausanne University Hospital and University of Lausanne Lausanne Switzerland

**Keywords:** culling, diet quantification, ecosystem management, environmental DNA, grazing ecosystems, niche overlap, rewilding

## Abstract

In tropical grazer assemblies with abundant large predators, smaller herbivores have been shown to be limited by predation and food quality, while the larger species are regulated by food abundance. Much less is known about herbivore resource partitioning in temperate grazing ecosystems, where humans typically regulate large animal abundances. The Oostvaardersplassen ecosystem in the Netherlands is a unique multispecies assemblage of cattle, horses, red deer, and geese developed after the initial introduction of a few individuals in 1983. During the first 35 years, this herbivore assemblage without predation or human regulation gradually changed into an increasing dominance of the smaller herbivore species. Carrying capacity was reached around 2008, after which numbers started fluctuating depending on winter conditions. From 2018, management changed and population numbers became regulated for biodiversity and animal welfare reasons; however, population numbers still remained close to carrying capacity for several years. We used eDNA metabarcoding of dung to quantify the diet composition of cattle, horses, red deer, and geese, annually in early winter from 2018 to 2021 and calculated their niche overlap. We found strong interspecific diet overlap. Horse and cattle diets remained mostly stable with fluctuating densities of the different species, while only red deer diet showed density dependence. Interspecific niche overlap decreased with increasing red deer population size, the most abundant species. When calculated as total energy expenditure, we found that niche overlap was more linked to red deer abundance than to total herbivore energy intake. We suggest that red deer changed their diet mainly in response to their own population size, reducing their niche overlap in relation to their population increase. In this case, resource competition reduced resource availability and forced herbivores to consume different plant taxa. We conclude that in this predator‐free temperate ecosystem, inter‐ and intraspecific resource competition are key factors structuring this assemblage of different size herbivores. We find a general competitive advantage of the more diet‐flexible red deer over horses and cattle, but with also clear signs of resource partitioning.

## INTRODUCTION

Terrestrial ecosystems with a dominant role of large herbivores are still widely found across the tropics, as in savannahs, but become increasingly restricted to smaller, often fenced areas due to the growing human population and associated land use changes (Olff et al., 2002). In Europe, by contrast, “grazing ecosystems” are being increasingly enlarged through “rewilding” initiatives, although they often lack top predators (Svenning et al., 2016) or opportunities for long‐distance seasonal migration due to barriers. These management interventions in these ecosystems are usually combined with removal, reintroduction, or culling, which potentially changes species' ecological roles, such as their diet selection and competitive and/or facilitative interactions, with potential cascading effects on other species groups (Danell et al., 2006; Pansu et al., 2019). Yet, the ecosystem‐wide consequences of different management interventions for large herbivore community dynamics in grazing ecosystems are still poorly understood. Studying the role of herbivores and their interactions in ecological networks can be done through the diet composition of each herbivore species, so the preference or avoidance of different plant taxa and the resulting diet overlap between different species can be assessed. This provides insights on intra‐ and interspecific interactions beyond studying population dynamics, and it can also inform on (density‐dependent) competition, resource partitioning (Kartzinel et al., 2015), or landscape usage (Abbas et al., 2011) among others.

The Oostvaardersplassen ecosystem (OVP), the Netherlands, has become a benchmark for rewilding in northern Europe (Gordon et al., 2021; ICMO2, 2010; Jepson, 2016; Lorimer & Driessen, 2014) and has served as a model temperate grazing ecosystem (Smit et al., 2015). The area, which is fully fenced, lacks top predators, making herbivore populations bottom‐up regulated (Frank et al., 2018). In the OVP, the absence of larger predators and of longer distance migration has created a system that is highly beneficial for smaller herbivores (Cornelissen, 2017). In the presence of long‐distance migration opportunities and large predators, multiple size classes are expected to dominate the herbivore assemblage, as seen in African grazing ecosystems (Fryxell & Sinclair, 1988; Owen‐Smith et al., 2020; Van Der Plas et al., 2016). As such, population growth was regulated by environmental conditions, resource availability, and interspecific competition until 2018.

**FIGURE 1:**
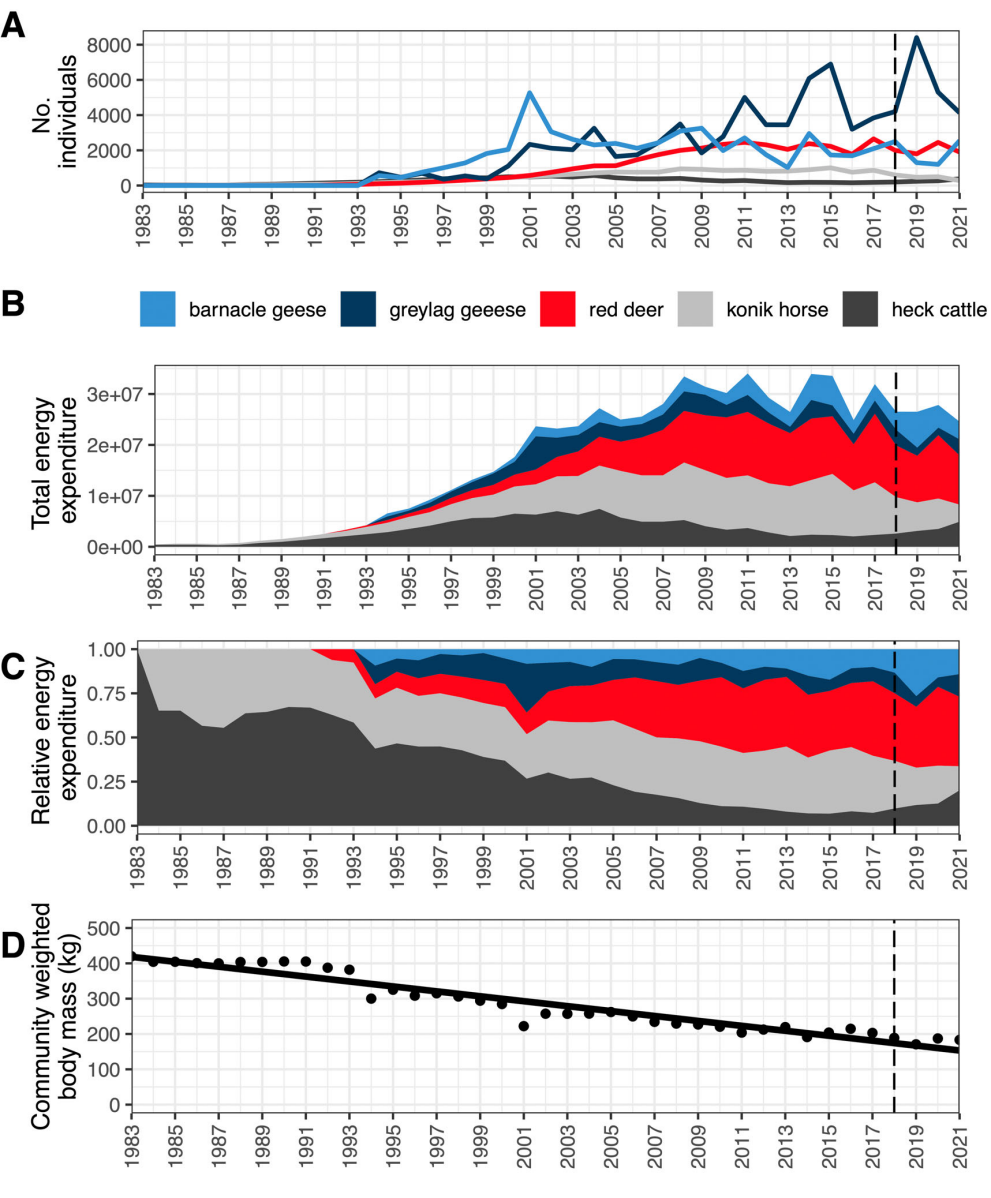
(A) Herbivore population size since 1983. (B) Total energy expenditure expressed as kilocalories per day. Each species' energy expenditure is represented as the width of their ribbon, not the total amount from 0 to the top of their ribbon. (C) Relative energy expenditure, calculated as the percentage of the total energy expenditure shown in (B). (D) Average body weight of one herbivore per year in the reserve. Notice the continuous decrease toward smaller herbivores throughout the years (*R* ^2^ = 0.92, *p*‐value < 0.001). Raw data available in Appendix S1: Table S2.

Since the reintroduction of red deer (*Cervus elaphus*) in 1992, this species has experienced a sustained population growth compared to the other large herbivores at the OVP (i.e., horse, *Equus ferus caballus* and cattle, *Bos taurus*; Figure 1A), which has steadily reduced the community weighted biomass (i.e., the mean weight of each herbivore; Figure 1D). Several conservation organizations (Buurmans, 2021; Theunissen, 2019) argued that the carrying capacity of the reserve had been reached, and hence, population numbers should be reduced by human intervention (Figure 1B,C). Other organizations also complained about the loss of biodiversity, especially birds, and intervention was advised from both an animal welfare and a biodiversity perspective. The severe climatic conditions of the area, the lack of sheltering landscapes, and resource limitation due to fencing or herbivore competition were some of the hypotheses to explain such mortality. Furthermore, both breeding and foraging habitat of protected bird species had decreased in the grazed area as a result of the changing grazed landscape under the high grazing pressure of the large herbivores (Mouissie et al., 2020). After two harsh winters (2016 and 2017), the area was severely exposed to outcries in the media about the increasing number of mortalities (especially horses, drawing public attention). Increasing presence on the news, public debates, and protests about animal welfare concerns (discussed in Theunissen, 2019) and the knowledge of the effects of the herbivores on protected bird species triggered a change in the management of the large herbivores. From 2018, all herbivore populations were actively regulated by culling, aiming at a target maximum density per species, in order to stop the decrease in biodiversity and to improve animal welfare. To achieve this, targets were to reduce (compared to carrying capacity) the population numbers by about 80% of the red deer and about 50% of the horse population. As the cattle population was below its target density in 2018, reduction was not necessary in the first 2 years. From 2020, cattle were also culled annually. The reduction in numbers was not achieved in 1 year, but took several years in which population numbers fluctuated. This intervention in a previously unregulated diverse community assembly provided a unique opportunity to study the effects of population size changes on their diet composition and niche overlap. See Figure 1A–C and Appendix S1 for further contextualization of the Oostvaardersplassen herbivore dynamics.

In this project, we collected dung of cattle, horses, red deer, and geese (both Greylag, *Anser anser*, and Barnacle geese, *Branta leucopsis*) in November 2018 before the culling and three times after the culling had started (November 2019, 2020, and 2021) to follow up potential associated changes in diet in response to both this management change and inter‐annual population fluctuations. We used environmental DNA (eDNA) metabarcoding to study the effect of density dependence on the niche overlap between the four main vertebrate herbivore species in the Oostvaardersplassen rewilding area. Metabarcoding of plant DNA present in the herbivores' dung allows for the estimation of the relative contribution of each plant taxon to their diet. This method allows for more precise taxonomic identification of plant taxa than microhistological techniques do (Valentini et al., 2009), unlocking a new approach in community ecology to study diet composition, species competition, and niche partitioning (Kowalczyk et al., 2011; Mas‐Carrió et al., 2024; Pegard et al., 2009; Rayé et al., 2011; Soininen et al., 2009).

The net consequences of density variation for resource partitioning within a large herbivore assemblage close to carrying capacity are still unclear. As such, the subject of investigation of this study was to explore whether culling and the associated changes in herbivore densities were linked with species‐specific diet shifts.

## MATERIALS AND METHODS

### Study area

The Oostvaardersplassen is a nature reserve in the center of the Netherlands (56 km^2^) (Appendix S1: Figure S1A). The Oostvaardersplassen was initially designated for industrial and agricultural purposes but became recognized as a key important breeding location for birds that were rare or absent in the Netherlands (Smit et al., 2015). In 1983, 32 Heck cattle were introduced, followed by 18 Konik horses in 1984 and 44 red deer in 1992. By May 2017, these populations had grown to 180 cattle, 864 horses, and 2650 red deer (Figure 1). These large herbivores are restricted to stay in the boundaries of the protected area by a fence and mostly use the grasslands, where there are no top predators. See Appendix S1 for further description of the Oostvaardersplassen ecosystem.

### Herbivore census

Herbivore populations have been counted on a yearly basis since 1983 (Appendix S1: Table S2). Populations were estimated by the management authority rangers through whole‐area ground counts from vehicles, complemented by aerial counts since 2011. Herbivores were counted each October and May to estimate the winter mortality and reproductive rates for each species. We used the counts from October. Geese populations (both barnacle and greylag) are calculated as the average number of wintering geese per month, counted from December through April.

### Plant surveys

The plant composition in the Oostvaardersplassen was determined using Braun‐Blanquet abundance categories (Braun‐Blanquet, 1932). This classification method groups plant taxa into nine different categories based on coverage ranges. We measured plant coverage this way using forty‐five 2 × 2 m quadrants on the grassland. This vegetation survey was conducted in August 2018 before the dung sampling began (Appendix S1: Table S1). Braun‐Blanquet categories were transformed to relative abundance, keeping the median value of the range of abundance for each category. We used the same plant composition of the Oostvaardersplassen for the four sampled years because the plant composition of the grassland remained stable (Cornelissen, 2017).

### Sample collection

Dung samples were collected across the grassland part of the OVP, with collections divided into three sub‐areas (see Appendix S1: Figure S1A) during November 2018, 2019, 2020, and 2021 for the four main herbivore species, that is, cattle, horses, red deer, and geese (Barnacle geese and Greylag geese combined as the species were not identified from the dung shape). Per species and year, 15 scat samples were collected (5 per sub‐area, Appendix S1: Figure S1A), leading to a total of 60 scat samples per year. Samples were spaced by at least 10 m to reduce the chance of re‐sampling the same individual, and GPS coordinates were taken for each sample. Only freshly deposited dung samples were collected. Freshness was assessed based on visual observation of deposition or on the dung moisture. In case of rainy conditions, old and fresh dung could be confounded. In that case, we avoided dung that was too wet (or with puddles within) and sampled only where the individuals had been within the same sampling day. Samples were then stored in dried silica beads at room temperature to dry and preserve them, without need for freezing, until DNA extraction could be done at the University of Lausanne, Switzerland.

### DNA extraction

We used between 0.5 and 1 g of dry dung as the starting point for the extraction. Extractions were performed using the NucleoSpin Soil Kit (Macherey‐Nagel, Düren, Germany) following the manufacturer's protocol. A subset of the extractions was tested for inhibitors with quantitative real‐time PCR (qPCR) applying different dilutions (2×, 10×, and 50×) in triplicates. qPCR reagents and conditions were the same as in DNA metabarcoding PCR reactions (see below), with the addition of 10,000‐fold diluted SybrGreen (Thermo Fisher Scientific, USA). Following these analyses, all samples were diluted fivefold before PCR amplification. All extractions were performed in a laboratory restricted to low DNA‐content analyses.

### DNA metabarcoding

DNA extracts were amplified using a generalist plant primer pair (Sper01; Taberlet et al., 2018), targeting all vascular plant taxa (Spermatophyta). Sper01 targets a 10‐ to 220‐bp gene fragment of the P6 loop of trnL intron, chloroplast DNA. To assign the DNA sequences to each sample, primers were tagged with eight variable nucleotides added to their 5′‐end with at least five differences between tags. The PCRs were performed in a final volume of 20 μL. The mixture contained 1 U AmpliTaq Gold 360 mix (Thermo Fisher Scientific), 0.04 μg of bovine serum albumin (Roche Diagnostics, Basel, Switzerland), 0.2 μM of tagged forward and reverse primers, and 2 μL of fivefold diluted template DNA. PCR cycling conditions were denaturation for 10 min at 95°C, followed by 40 cycles of 30 s at 95°C, 30 s at 52°C, and 1 min at 72°C, with a final elongation step of 7 min at 72°C. Amplifications were performed separately for each species and in replicates (4 per sample) in PCR plates each containing 60 DNA extracts, 12 blanks as well as 8 extraction, 8 negative, and 8 positive PCR controls (DNA assembly of 10 plant species with increasing relative concentrations). The use of blanks allows estimating the proportion of tag switches (i.e., false combination of tags, generating chimeric sequences) during library preparation (Schnell et al., 2015). Amplification success and fragment sizes were confirmed on agarose gel. PCR products were subsequently pooled per PCR plate. Amplicons were purified using a MinElute PCR Purification Kit (Qiagen, Hilden, Germany). Purified pools were quantified using a Qubit 2.0 Fluorometer (Life Technology Corporation, USA). Library preparation was done following the TagSteady Protocol (Carøe & Bohmann, 2020). After adapter ligation, libraries were validated on a fragment analyzer (Advanced Analytical Technologies, USA). Final libraries were quantified, normalized, and pooled before 150 paired‐end sequencing on an Illumina MiniSeq sequencing system with a Mid Output Kit (Illumina, San Diego, CA, USA).

### Bioinformatic data analyses

The bioinformatic processing of the raw sequence output was performed using the OBITools package (Boyer et al., 2016). Initially, forward and reverse reads were assembled with a minimum quality score of 40. The joined sequences were assigned to samples based on unique tag combinations. Assigned sequences were then de‐replicated, retaining only unique sequences. All sequences with fewer than 10 reads per replicate were discarded as well as those not fitting the range of metabarcode lengths. This was followed by two different clustering methods. First, pairwise dissimilarities between reads were computed, and lesser abundant sequences with single‐nucleotide dissimilarity were clustered into the most abundant ones. Second, we used the Sumaclust algorithm (Mercier et al., 2013) to further refine the resulting clusters based on a sequence similarity of 97%. It uses the same clustering algorithm as UCLUST (Prasad et al., 2015), and it is mainly used to identify erroneous sequences produced during amplification and sequencing, derived from its main (centroid) sequence. Remaining sequences were assigned to taxa using a reference database. We built a database for Sper01 by running an in silico PCR based on all the plant sequences available in the EMBL database (European Molecular Biology Laboratory). We kept a single sequence per taxonomic ID that was annotated at least to the genus level.

Further data cleaning and filtering was done in R (version 4.0.2) using the metabaR package (Zinger et al., 2021). Sequences that were more abundant in extraction and PCR controls than in samples were considered as contamination and removed. Operational taxonomic units (OTUs) with similarity to the reference sequence lower than 97% were also eliminated from the dataset. Removal of tag‐leaked sequences was done independently for each library. This approach allowed us to discard single OTUs instead of whole PCR replicates. However, PCR replicates with too small reads count were also discarded.

Remaining PCR replicates were merged by individual, keeping the presence–absence (P/A), frequency of occurrence (FOO), and mean relative read abundance (RRA). For studying plant selectivity, we used the RRA instead of using FOO or P/A because we were interested in the relative consumption of each plant taxon rather than which species are consumed. First, we calculated the dissimilarity matrix (Bray–Curtis distance) for each individual dung sample based on the final OTU table (for RRA, FOO, and presence–absence) and visualized the variation in OTU composition between individual dung samples using non‐metrical dissimilarity scaling using the FOO dataset (NMDS).

### Herbivore biomass and energy expenditure

To be able to compare species with different body mass, herbivore counts were transformed to herbivore biomass and daily energy expenditure (DEE, in kilojoules per day). Geese values used for the total energy expenditure correspond to the October counts. As the individual average body mass of each herbivore, we used: heck cattle (420 kg), konik horse (375 kg), red deer (120 kg), greylag geese (3.3 kg), and barnacle geese (1.9 kg). To calculate their population‐level energy expenditure, we use the allometric relation between individual body mass and metabolic rate. For this, we used Equation (1) for large herbivores (Demment & Van Soest, 1985) and Equation (2) for geese (Mooij, 1992):

(1) DEE=140×live body weight^0.75

(2) DEE=2.55×417×live body weight^0.71/2

Calculations for geese were done for barnacle and greylag geese independently. For the two geese species, the result was divided by two because they were estimated to spend approximately half their time foraging in the Oostvaardersplassen on an annual basis, compared to the large grazers that are there every day.

### Herbivore selectivity

We calculated the selectivity of the different herbivore species for each plant taxon by comparing the selected diets to the available diet (i.e., plant relative abundances in the field). We used Jacobs' *D* index (Jacobs, 1974) to measure plant selectivity, as follows:

(3) $$D=\frac{r-p}{r+p-2\mathrm{rp}}$$

*r* indicates the relative abundance of a plant taxon in the diet of an individual, and *p* indicates the relative abundance of that same plant taxon in the environment. *D* values range from −1 to 1. Negative values indicate avoidance, and positive values indicate selection. Values close to 0 indicate similar utilization to availability. We calculated Jacobs' *D* index and categorized each plant taxon as selected or not using only the RRA dataset; this is the case for all selectivity‐related calculations. When visualizing the Jacobs' *D* selectivity index, we also calculated the number of individuals for each year (up to 15) that ate each plant taxon. This indicates the spread of consumption of a plant taxon within each species.

### Niche overlap

We calculated Pianka's niche overlap index (Equation 4; Pianka, 1973) using the *spaa* package to investigate niche overlap within and between species as follows:

(4) $${\text{Pianka}}_{\mathrm{jk}}=\frac{\sum_{i}^{n}p_{\mathrm{ij}}p_{\mathrm{ik}}}{\sqrt{\sum_{i}^{n}p_{\mathrm{ij}}^{2}\sum_{i}^{n}p_{\mathrm{ik}}^{2}}}$$

where *p*_*ij*_ and *p*_*ik*_ are the proportion of plant OTU *i* by individual *j* and *k*, respectively, and *n* is the total number of plant OTU categories. Values close to 0 indicate no overlap and close to 1 indicate full overlap, that is, the same diets. All niche analyses were carried out using the FOO dataset.

Finally, we used generalized linear mixed models (GLMMs), with the *glmmTMB* package, to investigate the significance of niche partitioning for each species interactions across the sampled years. Data distribution was assessed using the *performance* package. We first explored the observed niche overlap for the 10 types of species interactions (i.e., all combinations of cattle, red deer, horse, and geese) for each different year. We used the Year variable instead of the separate counts of herbivores or their energy expenditure because Year condenses the fluctuations in herbivore population as a categorical variable. The model was built using a beta distribution and with the *glmmTMB* R package, as follows:

Model1:Niche overlap~Species interaction/Year

We also modeled niche overlap between species against herbivore energy expenditure (for all species together and separately) in order to scale the relative consumption of resources by each species:

$$\begin{array}{l} \text{Model}\;2:\text{Niche overlap}\sim \text{Species interaction}/\\ \text{Total energy consumption}+\left(1|\text{Year}\right) \end{array}$$

We further compared the Akaike information criterion (AIC), that is, relative quality of the statistical models for a given set of data, of each energy expenditure model (following the structure of Model 2) for all herbivores grouped (Total) and for each herbivore species individually.

See Appendix S1 for an overview of the decision‐making on data transformation for diet quantification.

## RESULTS

### Herbivore population dynamics

We visualized herbivore population dynamics in Figure 1A–D, showing the population by their energy expenditure, correcting for body size‐specific per‐mass energy use differences. They show ungulate populations in May (after winter mortality, before recruitment as the best stable estimate of long‐term population dynamics), but the analysis of the relations between density and diet was done using herbivore population sizes estimated in October (Appendix S1: Figure S1B), as they are more relevant for our diet analysis based on dung collected each November. Geese populations displayed in Figure 1 correspond to the wintering average. The long‐term population trends show a continuous increase in the red deer population (Figure 1A) until the reserve reached its carrying capacity (around 2011, Figure 1A,D). After this point, the total herbivore population started to fluctuate around its carrying capacity, but without a clear sign of decline after 2018 for the red deer, despite the change in management in that year. The horse population did decrease after 2018 as a result of the new management, and the cattle population first increased because of decreasing total large herbivore numbers in 2018 and 2019. This increase was also allowed because the number of cattle was below the target number. In 2021, the target number for cattle was reached, and from 2021, the cattle were also culled.

### Diet composition in relation to vegetation dominance

**FIGURE 2:**
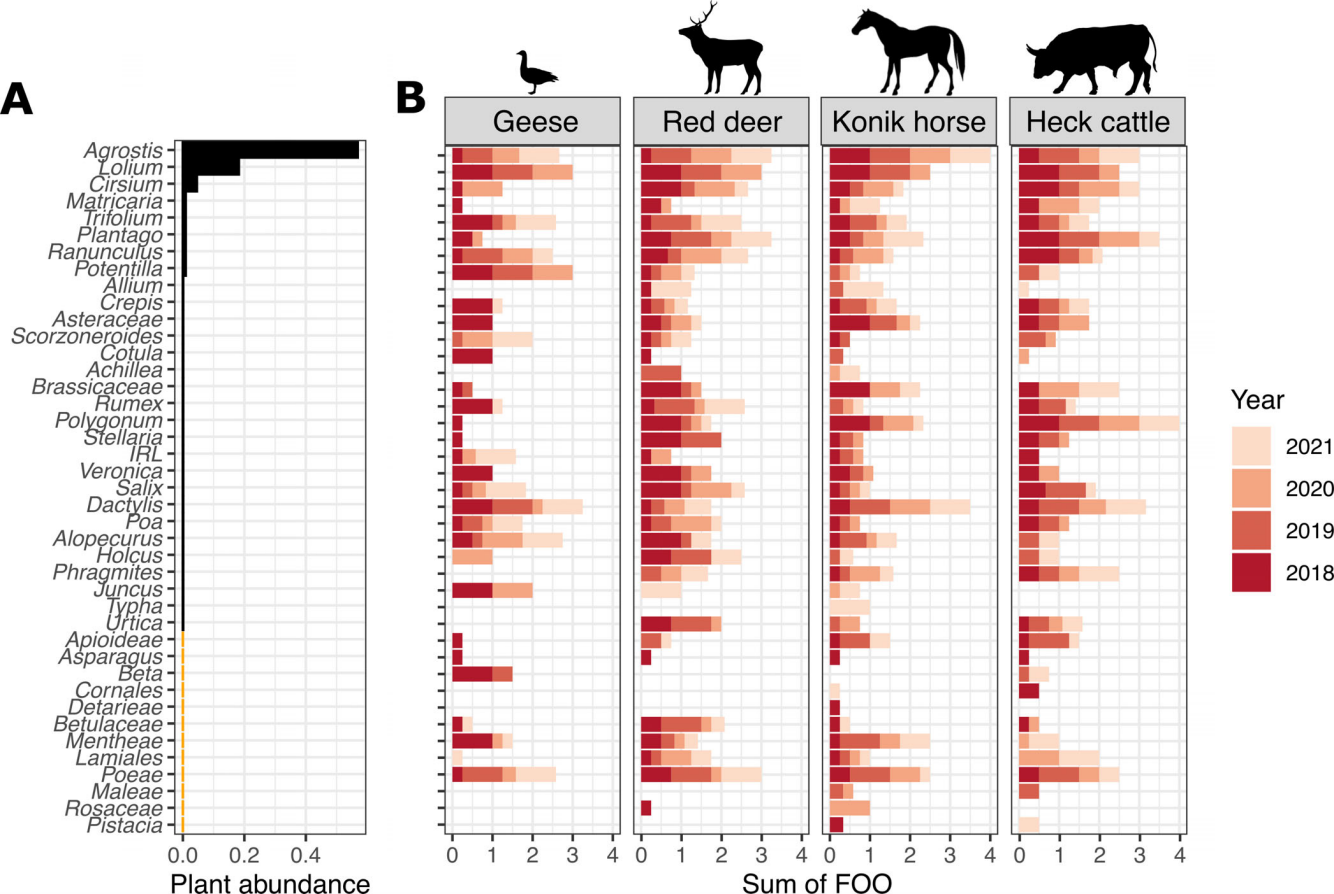
(A) Relative abundance in the Oostvaardersplassen ecosystem (OVP) per plant taxon and in the diet of the four herbivore species. Abundances shown in orange correspond to plant sequences which were not assigned to an existing species found in the Oostvaardersplassen. IRL stands for a subgroup of the *Fabaceae* family. (B) Sum of frequency of occurrence (FOO) for each species and year (see Appendix S1: Figure S2 for the sum of relative read abundance (RRA) for each species and year). Silhouettes obtained from Phylopic.org under public domain licenses (Anser, *Cervus elaphus*, and *Equus ferus caballus*: CC0 1.0 Universal Public Domain Dedication; *Bos primigenius taurus*: Public Domain Mark 1.0).

The grasslands of the Oostvaardersplassen were dominated by the graminoids *Agrostis stolonifera* and *Lolium perenne* and the forbs *Cirsium arvense* and *Plantago lanceolata* (Figure 2A). The diet analyses revealed substantial deviations from this, showing clear selection by the herbivores for a wider range of plant taxa. After quality filtering, we kept 3,472,674 reads of 284 different OTUs for the Sper01 assay that were assigned to 98 different plant taxa. We visualized the diet by plant genus for each species and year using the FOO data (Figure 2B, Appendix S1: Figure S2 using RRA). The dominant group was the *Poales* order (graminoids) for all herbivores, with *A. stolonifera*, *Dactylis glomerata*, and *L. perenne* as dominant species. This was expected given that the Oostvaardersplassen grasslands are mainly covered by graminoids. We also identified *Trifolium* sp. (Fabales) and *P. lanceolata* (Lamiales) as an important diet component, which are also relatively abundant in the grasslands (see Figure 2A, Appendix S1: Table S1 for the relative abundance of each plant taxon in the OVP).

### Herbivore diet composition

**FIGURE 3:**
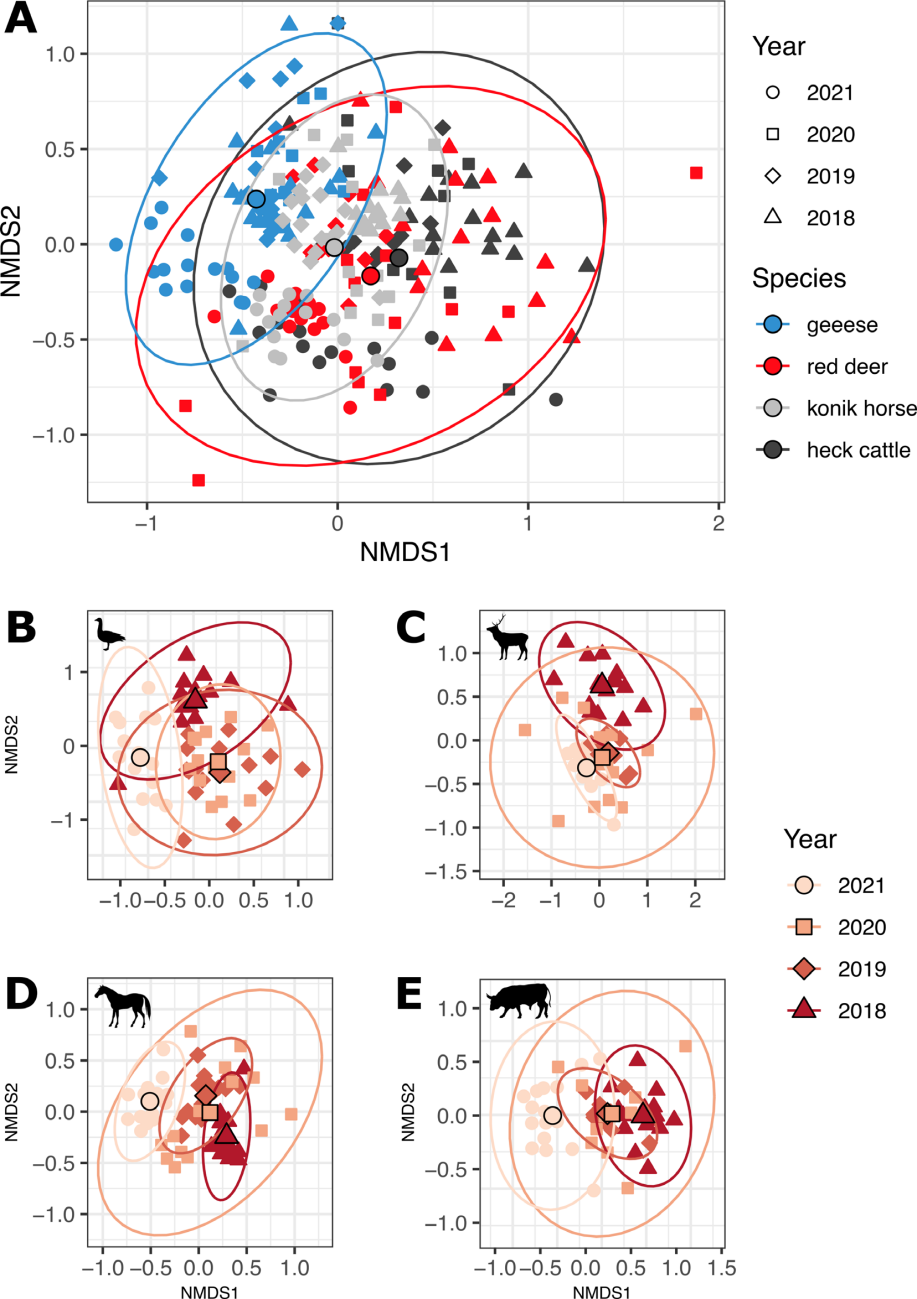
Non‐metrical dissimilarity scaling (NMDS) ordinations showing diet differences between species and years (A) and between years for each species (B–E). The NMDS calculation was done using the frequency of occurrence (FOO) data and including all the species together. (B–E) NMDS visualization calculated for each species separately. Points circled in black indicate the centroid for each group. Ellipses represent the 95% CI assuming a normal distribution of the data. Silhouettes obtained from Phylopic.org under public domain licenses (Anser, *Cervus elaphus*, and *Equus ferus caballus*: CC0 1.0 Universal Public Domain Dedication; *Bos primigenius taurus*: Public Domain Mark 1.0).

The NMDS was calculated including all species and years together to compare across species (Figure 3A, Appendix S1: Figure S3 for a comparison between RRA, FOO, and P/A). Horse diets clustered more than the other species did across the sampled years, and geese clustered the furthest from the other herbivores. Cattle and red deer diets mostly overlapped (Figure 3A). Horse multivariate space was in any case fully within the space of red deer and/or cattle. Moreover, substantial year‐to‐year differences were found in diet between herbivores, with an interaction between year and species (Figure 3B–E). By species, we observed that the diet of cattle and red deer clearly overlaps and has a similar range of variance. Furthermore, in 2020, the diets were the most dissimilar within each species, except for geese.

### Plant selectivity

**FIGURE 4:**
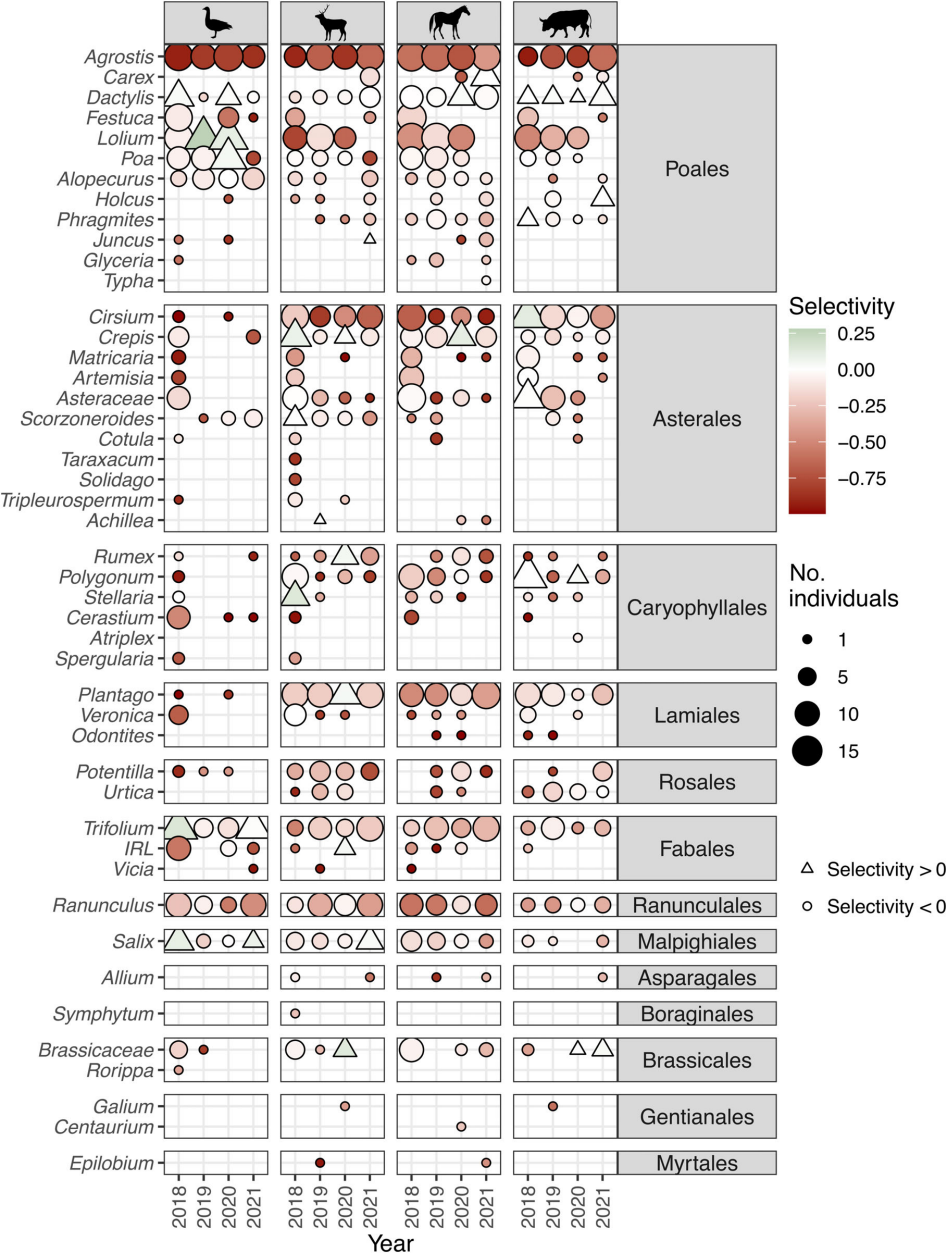
Selectivity for each herbivore species, year, and plant taxon which are found in the reserve using the relative read abundance (RRA) data. Size of the dots corresponds to the number of individuals (out of a maximum of 15) where the plant taxon was detected in their diet. Silhouettes obtained from Phylopic.org under public domain licenses (Anser, *Cervus elaphus*, and *Equus ferus caballus*: CC0 1.0 Universal Public Domain Dedication; *Bos primigenius taurus*: Public Domain Mark 1.0).

We further quantified the selectivity of each herbivore species (Equation 3, Figure 4). In general, the tendency of herbivores to avoid more common species was stronger than the tendency to select particular species, showing that the dominant species are not the most preferred ones. The most preferred plant taxa were not the same across herbivores. The most abundant plant taxon in the grassland, *A. stolonifera*, was not preferred by any of the species. Among asterales species, *Cirsium* sp. (likely *C. arvense*), was very abundant and was only selected by cattle in 2018. Interestingly, the forb *P. lanceolata*, which is also very abundant, was not selected by any herbivore species despite being found in their diets across the years.

### Niche overlap

**FIGURE 5:**
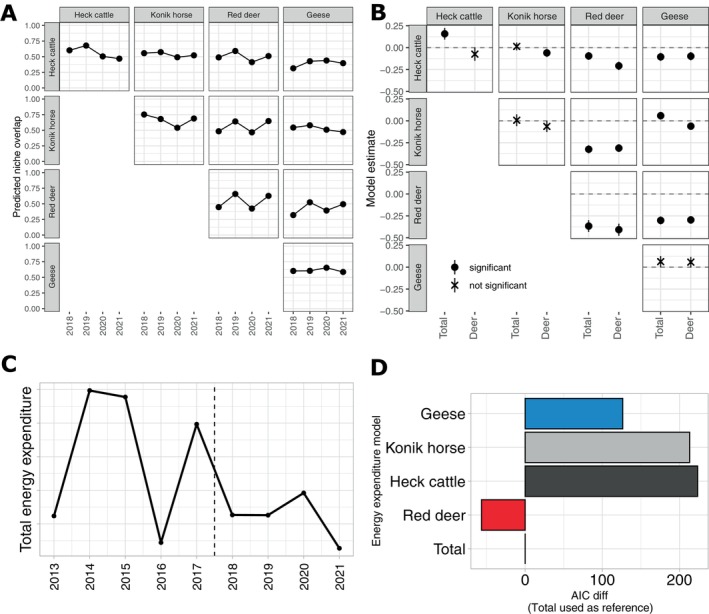
(A) Predicted niche overlap for each year and species interaction, extracted from Model 1. (B) Estimates of the regression line extracted from Model 2 for Total (all herbivore species together) and red deer, as those were the best‐performing models. (C) Detailed view of the total energy expenditure in the system during the last 10 years (see Figure 1B for the total energy expenditure since 1983). (D) Akaike information criterion (AIC) difference between models of niche overlap against energy expenditure. We use the model which accounts for the total herbivore energy expenditure as the zero reference. See Appendix S1: Table S3 for corrected *p*‐values for Figure 5B.

We then explored how the niche overlap between the four herbivores shifted between the herbivore species along the 4 years and how this related to the herbivore population shifts and year‐to‐year fluctuations in the study area. We calculated the niche overlap between each individual sample in order to test for the dietary niche overlap between and within species (using FOO). Only comparisons between individuals sampled during the same year were kept; that is, niche overlap comparisons across years were discarded. This model (Model 1) highlighted differences in predicted niche overlap between years for each combination of herbivore species (Figure 5A). We observed higher predicted niche overlaps in years 2 and 4, which align with the peaks of red deer population within the sampled years (Appendix S1: Figure S1B). To assess it, we ran linear correlations on each of the predicted niche overlaps from the aforementioned model predictors (Model 1) against the red deer numbers (Appendix S1: Figure S4A). Only red deer intraspecific niche overlap was significantly correlated to red deer population size. We also observed a marginal, non‐significant correlation both between red deer and horse and between red deer and cattle. However, this approach did not capture the fluctuations in the system as it only accounted for red deer numbers. To overcome this, we modeled the observed niche overlap against the total energy expenditure of herbivores in the system (i.e., population‐level energy expenditure, see Figure 5C for a detailed view of the energy expenditure in the system during the last 10 years) but also against each herbivore's population energy expenditure and extracted the estimates from each. We visualized the estimates of each model in Figure 5B (see Appendix S1: Table S3; Appendix S1: Figure S4B for all species comparisons). We extracted and visualized the AIC values of each version of Model 2 in Figure 5D to compare their performance depending on the species' energy expenditure included in the response variable. This served to test the effect each species population shift had on the niche overlap calculated.

We observe a clear similarity between the estimates for the total herbivore energy expenditure and the red deer energy expenditure, showing that the number of red deer influenced the diet selection of the other species. Both models outperformed those based solely on Heck cattle, Konik horse, or geese energy expenditure, as the latter had considerably lower AIC values. In particular, the estimates of total and red deer models (Figure 5B) are clearly negative for red deer‐related interactions, which aligns with the observed correlation of niche overlap being reduced with increasing red deer numbers (Appendix S1: Figure S4A). The results for geese–geese interactions are similar across models and non‐significant in all cases.

## DISCUSSION

For the past two decades, the Oostvaardersplassen ecosystem has been a hotspot of debate on grazing management between scientists, policy makers, managers, and members of the public, including citizen action groups (Theunissen, 2019), especially around the question whether management should interfere in herbivore population numbers by proactive culling to achieve the goals of biodiversity conservation of other species groups (insects, birds) and to answer societal animal welfare concerns. No interference in population regulation, that is, herbivores as an integral part of the ecosystem, would keep the populations bottom‐up regulated, with an increase in annual absolute mortality numbers due to lack of food and cast uncertainties in achieving conservation goals for specific birds and insects that better thrive at lower animal densities. In the popular media, the arguments switched between herbivore ecology (“Is regulation by food limitation in an incomplete system natural for large herbivores?”), ethical arguments (“We put them there in a small fenced area, so should we not take care of them?”), and biodiversity conservation arguments (“Decline of protected breeding and foraging birds, which need shrubs, taller herbs, and reed vegetation in their habitat, is not acceptable”). However, in the discussion on the consequences of food limitation, it remained unclear whether density‐dependent mortality mostly was arising from within‐species or between‐species density‐dependent population regulation. Nevertheless, in 2018, the management was changed from early reactive culling, that is, population dynamics controlled by food limitation, severity of winter and competition, and culling animals to prevent unnecessary suffering, to proactive culling to achieve the goals for biodiversity conservation and animal welfare.

Considering the herbivore population in terms of energy expenditure, the maximum was reached in 2011, at the same time as populations started to fluctuate from year to year in numbers (Figure 1A,B). Over time, the amplitude of the fluctuations seemed to increase. A similar pattern has been found for the long‐term feral population of sheep on St. Kilda island (Scotland), where the sheep population encounters no predators but limited food resources. Their population size oscillates around carrying capacity (Boyd et al., 1964; Crawley et al., 2021). However, this system is composed of a single herbivore species. In the OVP, we found that declines in one species were typically not compensated by an increase in another species, suggesting constraints from winter conditions, with the exception of the relative increase of about 50% in cattle since the start of active population regulation in 2018. This rise could also suggest an intermediate feeding strategy in cattle, that is, they have a varied diet as red deer did, but red deer are better competitors in a forage‐limited system due to their narrower mouths and smaller size (Latham et al., 1999). However, when released from competition with red deer and horses, cattle can recover because they deal with coarse, low‐quality food better through a greater harvesting rate (Dinius & Krause, 1965). Moreover, cattle benefited from taller grass thanks to the red deer and horse removal, leading to higher survival and population rise.

Despite the introduction of proactive culling, the red deer population was not steadily reduced likely because of their high reproductive rate, which also kept herbivore numbers at carrying capacity from 2008 to 2017 (Figure 1B,D). They also used the marshland as a foraging and resting habitat, which is difficult to access by managers and complicates proactive culling. But the whole herbivore community energy expenditure did not change much (Figure 1B), implying that our diet studies addressed the resource partitioning of different‐sized herbivores close to carrying capacity in relation to year‐to‐year population fluctuations. The main herbivore biomass shift from 2018 to 2021 was that cattle became relatively more abundant than horses (Figure 1C).

In ecosystems with larger predators, we expect smaller herbivores to be limited by predation and larger species by food quality and quantity (Hopcraft et al., 2010). Without predation, as in the Oostvaardersplassen, smaller herbivores are expected to outcompete larger ones due to higher population growth rates (faster recovery after strong winters) and their capacity to graze the grass too short for large species. The long‐term population data of Oostvaardersplassen support this expectation as the mean body size of the assembly consistently declines over time (Figure 1D).

### Plant selectivity

Interestingly, the most abundant plant taxa in the grassland were not the most preferred ones by the herbivores (Figure 4), despite being consumed by the majority of them. Herbivores tend to avoid the most common taxa rather than selecting particular ones. This is a typical pattern for intensely grazed systems: the preferred plant species will decline in abundance due to consumption, making them rarer, leaving the less preferred species to dominate (Török et al., 2018). This tendency can lead to the point where the preferred plant species become more difficult to find, ultimately decreasing their preference by herbivores. This could explain why in the Oostvaardersplassen, grazing intensity has driven herbivores to accommodate their diet for the more common species, without really preferring them at lower herbivore population densities. Examples are thistles (Asterales) and reeds (*Phragmites* sp.), taxa with low preference, which are equally spread in the diet of all herbivores except geese, which fully avoid them. Since geese can reach pastures outside the fenced area, they are less influenced by the herbivore densities in the reserve, allowing them to be more selective for highly palatable plant taxa compared to land herbivores.

Furthermore, the selectivity trend observed for *D. glomerata* in cattle suggests that their higher biomass density makes it more interesting to increase their instantaneous intake rate. Overall, the consistency across years validates our methodology and provides a detailed view of the dietary choice of each herbivore species.

When red deer population size was greater (i.e., 2018 and 2020), we detected lower niche overlap results for these years (Figure 5A), suggesting that their feeding strategy changed to include a greater variety of plant species. Since the total herbivore energy expenditure remained high and relatively stable along the sampled years (Figure 5C), we could be observing the result of facilitation, where red deer would have homogenized vegetation composition toward a higher quality one, benefiting other herbivores. However, we are unable to disentangle whether this feeding shift was a consequence of the red deer population reduction, which could have led to an increased vegetation height allowing cattle to better select among them, or due to a behavioral shift because of the culling disturbance. In other words, culling of red deer individuals triggered further dispersion, and thus, they fed on a greater diversity of plant taxa, possibly beyond the dry grassland part and into the marsh of the OVP. This could be because from 2018 the water level in the marsh was lowered for the regeneration of reed vegetation, and on the emerging bare soils, extensive pioneer vegetation developed. Such explanation falls in the landscape of fear domain (McArthur et al., 2014), that is, red deer increasing their time in the marsh area because they are less likely to be culled than in the grassland. Unfortunately, we do not have data on the plant composition of the marsh part or on the proportion of time red deer spent in the grassland and the marsh across the sampled years, but they were also utilizing resources from the marsh as part of this behavioral response (personal observation). An alternative explanation could be that culling pressure led to a lower selectivity of plant taxa, and thus, their diet became more diverse as they put less effort in the selection. However, if that was the case, we should have observed a decrease in their selectivity index. In any case, we suggest that this approach (Figure 4) could be used to monitor herbivory efficiency and is particularly suitable for within‐species comparisons or between herbivore species with similar digestive systems.

### Niche overlap

The diet of the three ungulate species in November was quite similar to that seen from the NMDS plots and the niche overlap calculations, suggesting overall strong resource overlap between these species of very different body mass. However, they differ on the least abundant plant taxa, which align to the NMDS. Cattle and red deer (both ruminants) consume less abundant plants equally compared to horse and geese (both non‐ruminants), which differ from the former two but do not group together. Our results suggest that horses rely heavily on a few plant taxa in November, which are also eaten by red deer and cattle (full overlap in the NMDS, Figure 3A). The close distribution of individual horses in the NMDS (Figure 3A and Appendix S1: Figure S3), which move in a single herd, suggests that horses had a homogeneous diet composition across individuals compared to red deer. This could indicate greater competition within the horse population to get resources but also lesser competition for cattle and especially red deer because of their greater choice of plant taxon. Also, it suggests that digestive strategy (Iason & van Wieren, 1999) is more important in this system in this period of the year than body size to understand niche overlap. Geese have the least overlapping diets among the sampled species (Figure 3A). They are the least influenced by herbivore densities or plant availability in the Oostvaardersplassen. In fact, their intraspecific niche overlap was the most stable across the sampled years (Figure 5A), suggesting that their dietary choices were not influenced by culling.

We found that niche overlap during the sampled period decreases when total or red deer energy expenditure increases (Figure 5B). This likely reflects the relative impact of red deer population size on the total energy expenditure and the ecosystem as a whole and suggests that red deer responded to their population size increase in terms of diet, leading to more dissimilar diets at higher abundance and thus lesser niche overlap. However, as culling and lowering the water level in the marsh were also happening at the same time, it is difficult to distinguish between these factors and which ones had the highest impact. Contrastingly, they are the smallest of the three land herbivores and may then be superior competitors (e.g., by being able to graze preferred plants the shortest) forcing larger species to select other, less favorable resources. This may support the idea that in the absence of predation regulation, smaller species are more limited by food quality and larger species by food quantity (Bukombe et al., 2019; Skogland, 1991). But this also may reflect that red deer, being a mixed feeder on grasses, forbs, and woody species, are simply more flexible to shift their diet than grazers such as horses and cattle.

## CONCLUSIONS

Our results indicate that culling of red deer coincides with more dissimilar diets for cattle and red deer in early winter. However, the total herbivore density did not change much (Figures 1B,C and 5C). In the case of the OVP, decreasing the number of red deer by culling had no clear effect on shifting the diet composition of the other species. Rather, it was the individual variation in diets within red deer that responded the most to red deer numbers, with individuals selecting more dissimilar diets in November when red deer numbers were highest (Figure 5A).

Finally, it should be noted that the extensive annual proactive culling introduced in the Oostvaardersplassen since 2018 was not motivated in order to restore a competitive balance between different‐sized species or mimicking the effect of natural predation. Instead, the change in management was motivated by the impact of the large herbivores on biodiversity and for animal welfare reasons. Balancing different aspects of the management, whether or not to interfere in population dynamics of large herbivores and, in the future, also large predators, remains a challenge, including for the many new areas across Europe where a rewilding approach with large herbivores is now becoming adopted (Saavedra et al., 2023).

## AUTHOR CONTRIBUTIONS

Eduard Mas‐Carrió, Han Olff, and Luca Fumagalli designed the study and supervised all the analyses. Perry Cornelissen conducted the plant survey and supervised herbivore counting. Eduard Mas‐Carrió conducted the fieldwork, laboratory work, bioinformatics, data analyses, and prepared the figures. Eduard Mas‐Carrió wrote the first draft of the manuscript, with input from all other authors.

## CONFLICT OF INTEREST STATEMENT

The authors declare no conflicts of interest.

## Supporting information


Appendix S1.

## Data Availability

Data (Mas‐Carrió, 2024) are available in Dryad at https://doi.org/10.5061/dryad.prr4xgxth.
