# Supplementary material for: Density‐dependent resource partitioning of temperate large herbivore populations under rewilding
Source: Ecol Appl. 2025 Sep 15;35(6):e70090. doi: 10.1002/eap.70090 (PMC12434437; doi:10.1002/eap.70090)
Supplement: Supplementary file 1 — Appendix S1. [file EAP-35-e70090-s001.pdf]

Density-dependent resource partitioning of temperate large herbivore populations under rewilding

Eduard Mas-Carrió, Perry Cornelissen, Han Olff, and Luca Fumagalli

Ecological Applications

Appendix S1

Study area and herbivore population background

The Oostvaardersplassen is a nature reserve in the centre of the Netherlands (56 km^2) (Figure S1A). The area is situated at 4m below the water level of the surrounding Lake Markermeer, and emerged in 1968 as part of Zuidelijk Flevoland, a large land reclamation polder of 430 km2. The Oostvaardersplassen part of the polder was initially designated for industrial and agricultural purposes but became rapidly recognized as a key important breeding location for birds that were rare or absent at that moment in the Netherlands, such as greylag goose, spoonbill and bearded reedling (Smit et al., 2015). In fact, greylag geese started playing a key role in keeping the young developing reed marsh open, promoting the diversity of other wetland birds. In 1973-75, agricultural and industrial development was put on hold and nature-oriented water management was started to preserve and stimulate the marshes ecological value. Also neighbouring grasslands that were initially drained and developed for agriculture were added to the protected areas to support moulting greylag geese that played such a key role in the marsh part of the ecosystem, wintering geese, egrets, harriers, and other protected species that use this drained area as foraging habitat. This led to an ecosystem configuration of the Oostvaardersplassen of approximately half marshes dominated by *Phragmites australis* and half drained area with grasslands, tall herbs and reed vegetation scrub and forests. To keep these grasslands open and suitable for the geese, large herbivores were introduced 8 years later under a free-ranging management without interventions in their population development. In 1983, 32 Heck cattle were introduced, followed by 18 Konik horses in 1984 and 44 Red deer in 1992. By May 2017, these populations had grown to 180 cattle, 864 horses and 2650 red deer. These large herbivores are restricted to stay in the boundaries of the protected area within a fence and mostly use the grasslands, although some of the red deer also spend time in the marshes. In addition, the area saw a growing visitation by greylag geese, breeding, foraging and moulting in the marsh part, while also foraging in the grassland part; and barnacle and

white fronted geese, which visit the grasslands for foraging. There is some predation of red fox and white tailed eagle on geese, but without a large role in population regulation, while red deer, horses and cattle are not subject to predation. As for top predators, after an absence of 120 years, wolves returned to the Netherlands in 2018 but due to landscape fragmentation and lack of corridors this species has not reached the Oostvaardersplassen yet.

With the start of the proactive culling, a maximum capacity of 1100 mammalian large herbivores was set for the reserve, and population size of the three major herbivore species was planned to be reduced accordingly, making biodiversity, including protected bird species, and animal welfare the main focus of the conservation efforts (Begeleidingscommissie Beheer Oostvaardersplassen, 2018).

Herbivore size, food limitation and the role of predators

In ecosystems with a large role of big predators, smaller herbivores have been found to be more limited by predation than larger species (Sinclair et al., 2003), reducing their competition for food with large herbivores, where smaller species are more limited by food quality and larger species are limited by food quantity, linked to grass height (Hopcraft et al., 2010, 2012). In the fenced OVP trophic rewilding regime, without a large predators, previous studies found that increasing population sizes of all herbivore species lead to shorter grass but of higher quality (Cornelissen, 2017), leading to an expected competitive advantage of smaller herbivores and potential higher niche overlap between all species. However, these smaller species have less body reserves, making them potentially more sensitive to winter mortality, leading to stronger population fluctuations close to carrying capacity than in larger species, potentially relaxing competitive interactions (Coulson et al., 2001). Moreover, the different digestive strategies within the present herbivores, i.e. ruminants (Heck cattle and Red deer) and non-ruminants (Konik horse and Geese), determine how plant material is fermented and energy extracted and could also drive such competitive interactions (Clauss et al., 2003).

Due to the absence of predators in the OVP, trophic interactions are restricted to plant-herbivore interactions, i.e. diet quantification; and herbivore-herbivore interactions, i.e. niche overlap. Both metrics are based on the quantification of plant composition in each individual diet and in the environment. The OVP is a well-studied ecosystem and has a relatively low herbivore and plant diversity, which makes it technically easy to study. Sampling in such environments is not expensive nor time consuming, and the results can be compared to similar ecosystems across Europe.

Different hypotheses are possible for how interspecific niche overlap of different-sized herbivores changes with densities of an assemblage with species of different body size close to carrying capacity. On the one hand, increasing density may increase competition for preferred plants. Smaller species may then be superior competitors (e.g. by being able to graze preferred plants the shortest) which forces larger species to select other resources. This may indicate that smaller species are more limited by food quality and large species by food quantity. In that case higher total densities, especially of smaller species, are expected to reduce niche overlap. On the other hand, high population densities of all herbivores can reduce vegetation heterogeneity, and change heterogeneous vegetation to homogeneous, high quality sward that is beneficial of all species (facilitation effects). In that case, increasing density is expected to promote resource overlap.

Nevertheless, proactive herbivore culling can change species interactions and therefore potentially have a strong impact on biodiversity, productivity, nutrient cycling and soil health (Ripple & Beschta, 2004; Thoresen et al., 2021).

Herbivore biomass and energy expenditure

To be able to compare species with different body mass, herbivore counts were transformed to herbivore biomass and daily energy expenditure (DEE). Geese values used for the total energy expenditure correspond to the October counts. As individual average body mass of each herbivore we used: heck cattle (420kg), konik horse (375kg), red deer (120kg), greylag geese (3.3kg) and barnacle geese (1.9kg). To calculate their population-level energy expenditure, we use the allometric relation between individual body mass and metabolic rate. For this we used *Equation S1* for large herbivores (Demment & Van Soest, 1985) and *Equation S2* for geese (Mooij, J.H., 1992):

$$DEE = 140 * (\text{live body weight})^{0.75} \qquad \text{Equation S1}$$

$$DEE = 2.55 * 417 * (\text{live body weight}^{0.71}) / 2 \qquad \text{Equation S2}$$

Calculations for geese were done for barnacle and greylag geese independently. For the two geese species, the result was divided by two because they were estimated to spend approximately half their time foraging in the Oostvaardersplassen on an annual basis, compared to the large grazers that are there every day.

Considerations on data transformation for diet quantification

We used frequency of occurrence (FOO) instead of presence-absence (P/A) or relative read abundance (RRA) because it provides a balanced ecological signal between a qualitative (P/A) and quantitative approach (RRA) to study niche overlap from a dietary perspective (see Figure S3 for a visual comparison of the NMDS with the three data transformations). It accounts for the different plant taxon consumed by herbivores and the digestion, extraction, amplification and sequencing drawbacks (Deagle et al., 2019). Given this data is calculated from FOO, we minimise the effect of the digestive differences between herbivores. When visualising the FOO of each plant taxon found in the OVP (Figure 2B) we found that the most abundant plant taxons (Figure 2A) are consumed by the four herbivore species in all the sampled years.

However, the selectivity of each plant taxa was based on the RRA data instead of the FOO data because the latter is not a good proxy to infer relative plant consumption (Figure 4). Using the RRA implies that the results are sensitive to the herbivores' digestive biology, so only within-species comparisons are free of this bias. Differences in digestive tract morphology and functioning alters how plant material is broken down and impacts on the amount of plant DNA released during the DNA extraction (Kartzinel et al., 2015; Stapleton et al., 2022).

Methodological consideration on the sampling time

Our dataset was limited to samples collected in November, when the food availability is at its lowest. Sampling year-round, especially when conditions are milder, may further inform on the complete dynamics at the OVP, and put in perspective the results from November samples discussed here. Due to technical and legal constraints, the sampling could only be done in November the first two years. Thus, this sampling time was also maintained for the last two years.

Supplementary Figures

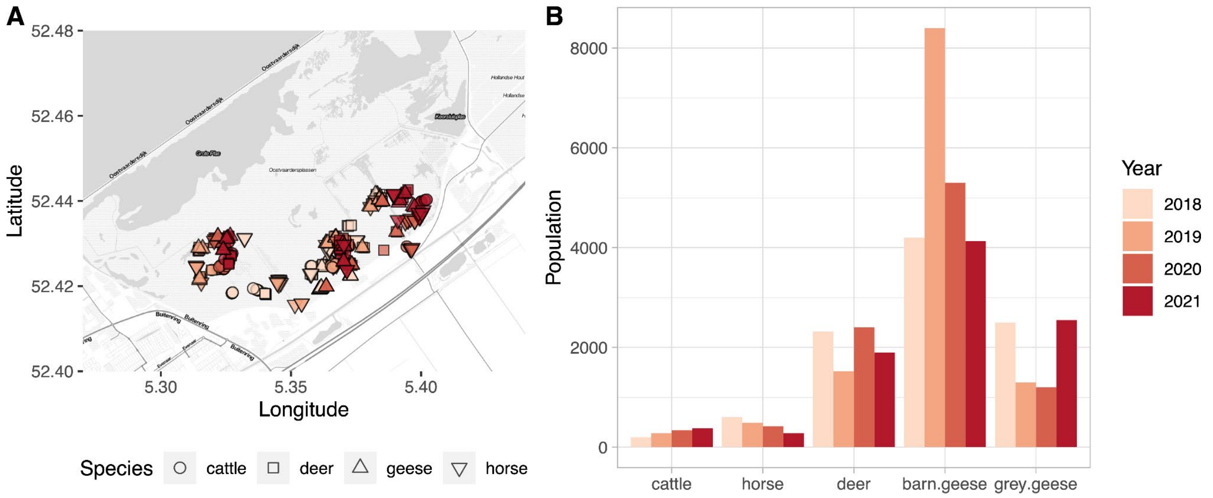

Figure S1. A) Map of the study area. Points indicate the location where each scat sample was collected for each species and year of the grassland. B) Herbivore population numbers for each of the sampled years.

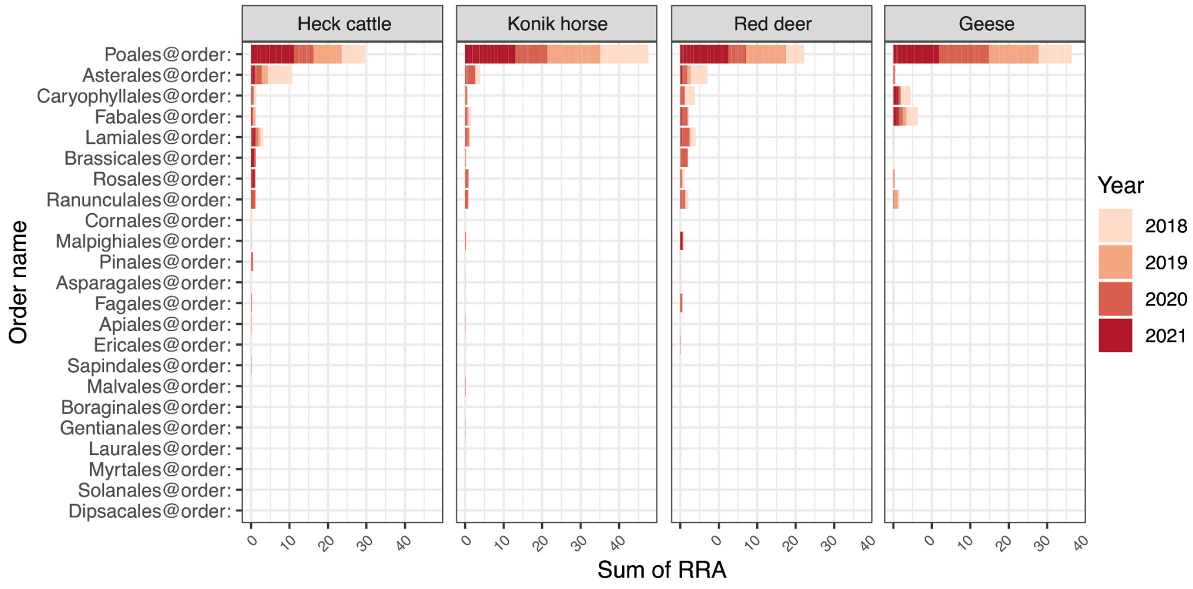

Figure S2. Barplot of Sum of RRA for each plant order, herbivore species and year.

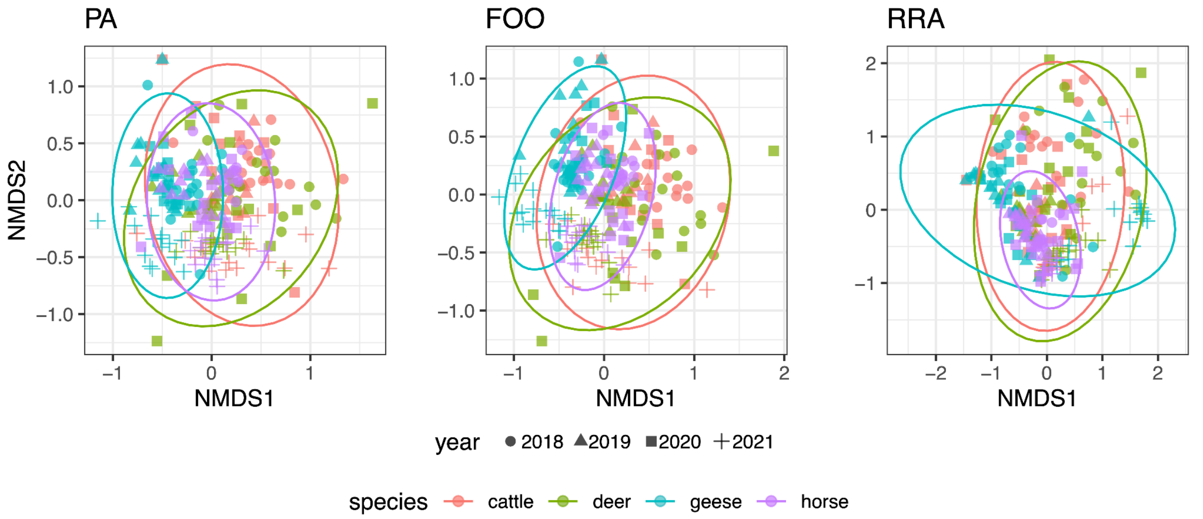

Figure S3. NMDS comparing the three data transformation methods. PA stands for presence/absence. FOO for frequency of occurrence. RRA for relative read abundance.

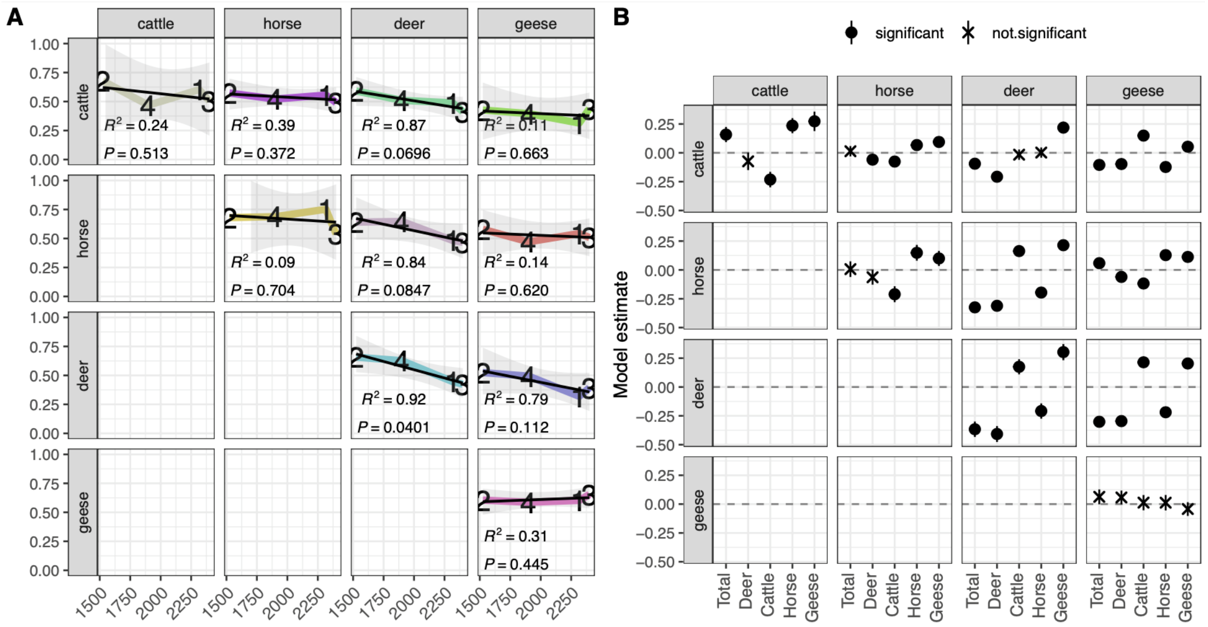

Figure S4. A) Linear correlation between the predicted niche overlap from model 2 and red deer population. R2 and p-values are indicated for each species interaction. The bold numbers indicate the year of sampling, 1 being 2018 and 4, 2021. B) Estimates for all species models including total (Figure 5 includes only Total and Red deer). Shape indicates if the result is significant or not. Dashed line indicates the 0.

Table S1. Plant species abundance at the OVP. Genus name and Order name are also provided. Abundance is shown in percentage, extracted as mean from the Braun-Blanquet categories.

Plant species	Abundance	Genus	Order
Agrostis stolonifera	56.8698817	Agrostis	Poales
Lolium perenne	18.1983621	Lolium	Poales
Plantago lanceolata	4.54959054	Plantago	Lamiales
Cirsium arvense	4.54959054	Cirsium	Asterales
Potentilla anserina	0.90991811	Potentilla	Rosales
Ranunculus repens	0.90991811	Ranunculus	Ranunculales
Odontites vernus subsp. serotinus	0.90991811	Odontites	Lamiales
Plantago major subsp. major	0.90991811	Plantago	Lamiales
Trifolium dubium	0.90991811	Trifolium	Fabales
Trifolium repens	0.90991811	Trifolium	Fabales
Cerastium fontanum subsp. vulgare	0.90991811	Cerastium	Caryophyllales
Cirsium vulgare	0.90991811	Cirsium	Asterales
Jacobaea vulgaris	0.90991811	Jacobaea	Asterales
Matricaria chamomilla	0.90991811	Matricaria	Asterales
Convalvulus sepium	0.09099181	Convalvulus	Solanales
Urtica dioica	0.09099181	Urtica	Rosales
Ranunculus acris	0.09099181	Ranunculus	Ranunculales
Ranunculus sceleratus	0.09099181	Ranunculus	Ranunculales
Bromus hordeaceus	0.09099181	Bromus	Poales
Echinocloa crus-galli	0.09099181	Echinocloa	Poales
Alopecurus geniculatus	0.09099181	Alopecurus	Poales
Dactylis glomerata	0.09099181	Dactylis	Poales
Eleocharis palustris	0.09099181	Eleocharis	Poales
Elymus repens	0.09099181	Elymus	Poales
Festuca rubra	0.09099181	Festuca	Poales
Holcus lanatus	0.09099181	Holcus	Poales
Juncus articulatus	0.09099181	Juncus	Poales
Juncus bufonius	0.09099181	Juncus	Poales
Phleum pratense	0.09099181	Phleum	Poales
Phragmites australis	0.09099181	Phragmites	Poales
Poa annua	0.09099181	Poa	Poales
Poa pratensis subsp. pratensis	0.09099181	Poa	Poales
Scirpus sp.	0.09099181	Scirpus	Poales
Typha	0.09099181	Typha	Poales
Carex	0.09099181	Carex	Poales
Glyceria	0.09099181	Glyceria	Poales
Epilobium parviflorum	0.09099181	Epilobium	Myrtales
Epilobium tetragonum	0.09099181	Epilobium	Myrtales
Salix alba	0.09099181	Salix	Malpighiales
Ajuga reptans	0.09099181	Ajuga	Lamiales
Glechoma hederacea	0.09099181	Glechoma	Lamiales

Limosella aquatica	0.09099181	Limosella	Lamiales
Mentha aquatica	0.09099181	Mentha	Lamiales
Veronica arvensis	0.09099181	Veronica	Lamiales
Veronica catenata	0.09099181	Veronica	Lamiales
Veronica persica	0.09099181	Veronica	Lamiales
Lycopus europeus	0.09099181	Lycopus	Lamiales
Geranium dissectum	0.09099181	Geranium	Geraniales
Galium	0.09099181	Galium	Gentianales
Centaurium pulchellum	0.09099181	Centaurium	Gentianales
Vicia	0.09099181	Vicia	Fabales
IRL	0.09099181	IRL	Fabales
Trifolium fragiferum	0.09099181	Trifolium	Fabales
Trifolium pratense	0.09099181	Trifolium	Fabales
Equisetum arvense	0.09099181	Equisetum	Equisetales
Spergularia	0.09099181	Spergularia	Caryophyllales
Atriplex prostrata	0.09099181	Atriplex	Caryophyllales
Persicaria lapathifolia	0.09099181	Persicaria	Caryophyllales
Persicaria maculosa	0.09099181	Persicaria	Caryophyllales
Polygonum aviculare	0.09099181	Polygonum	Caryophyllales
Rumex conglomeratus	0.09099181	Rumex	Caryophyllales
Rumex crispus	0.09099181	Rumex	Caryophyllales
Rumex maritimus	0.09099181	Rumex	Caryophyllales
Rumex obtusifolius	0.09099181	Rumex	Caryophyllales
Stellaria aquatica	0.09099181	Stellaria	Caryophyllales
Stellaria media	0.09099181	Stellaria	Caryophyllales
Brassicaceae	0.09099181	Brassicaceae	Brassicales
Brassica nigra	0.09099181	Brassica	Brassicales
Capsella bursa-pastoris	0.09099181	Capsella	Brassicales
Rorippa palustris	0.09099181	Rorippa	Brassicales
Sisymbrium officinale	0.09099181	Sisymbrium	Brassicales
Symphytum	0.09099181	Symphytum	Boraginales
Solidago	0.09099181	Solidago	Asterales
Asteraceae	0.09099181	Asteraceae	Asterales
Achillea millefolium	0.09099181	Achillea	Asterales
Artemisia vulgaris	0.09099181	Artemisia	Asterales
Bellis perennis	0.09099181	Bellis	Asterales
Bidens cernua	0.09099181	Bidens	Asterales
Bidens tripartita	0.09099181	Bidens	Asterales
Carduus crispus	0.09099181	Carduus	Asterales
Cotula coronopifolia	0.09099181	Cotula	Asterales
Crepis capillaris	0.09099181	Crepis	Asterales
Erigeron canadensis	0.09099181	Erigeron	Asterales
Eupatorium cannabinum	0.09099181	Eupatorium	Asterales
Gnaphalium uliginosum	0.09099181	Gnaphalium	Asterales
Pulicaria dysenterica	0.09099181	Pulicaria	Asterales
Pulicaria vulgaris	0.09099181	Pulicaria	Asterales

Scorzoneroides autumnalis	0.09099181	Scorzoneroides	Asterales
Sonchus arvensis	0.09099181	Sonchus	Asterales
Sonchus asper	0.09099181	Sonchus	Asterales
Taraxacum officinale	0.09099181	Taraxacum	Asterales
Tephroseris palustris	0.09099181	Tephroseris	Asterales
Tripleurospermum maritimum	0.09099181	Tripleurospermu m	Asterales
Tussilago farfara	0.09099181	Tussilago	Asterales
Allium	0.09099181	Allium	Asparagales
Daucus carota	0.09099181	Daucus	Apiales
Alisma lanceolatum	0.09099181	Alisma	Alismatales
Alisma plantago-aquatica	0.09099181	Alisma	Alismatales
Lemna minor	0.09099181	Lemna	Alismatales
Triglochin palustris	0.09099181	Triglochin	Alismatales

Table S2. Herbivore counts at the OVP in May for each sampled year.

year	cattle	horse	deer	barnacle geese	greylag geese
1983	32				
1984	31	18			
1985	31	18			
1986	24	20			
1987	32	28			
1988	58	36			
1989	75	45			
1990	102	54			
1991	130	70			
1992	160	86	40		
1993	190	120	63		
1994	221	156	104	726	562
1995	269	198	133	471	455
1996	319	222	181	693	759
1997	385	281	242	357	1011
1998	434	329	307	546	1285
1999	442	377	378	380	1818
2000	500	448	457	1106	2049
2001	486	499	571	2337	5265
2002	539	572	750	2122	3057
2003	485	637	949	2035	2624
2004	573	712	1118	3253	2307
2005	442	768	1126	1647	2393
2006	379	763	1466	1745	2120
2007	379	763	1758	2450	2437
2008	404	947	1997	3495	3090
2009	311	921	2126	1854	3256
2010	258	852	2340	2778	1987
2011	283	867	2454	5003	2706

2012	216	808	2311	3453	1749
2013	163	818	2055	3449	1025
2014	183	899	2378	6086	2952
2015	177	1006	2229	6898	1735
2016	157	758	1778	3197	1692
2017	180	865	2650	3844	2096
2018	200	600	2000	4200	2500
2019	240	470	1800	8400	1300
2020	270	500	2450	5300	1200
2021	380	280	1900	4131	2548

Table S3. Energy model estimates, std. error and p-values. Complementary information to Figure 5B. P-value correction was done using Bonferroni method. In 8 out of 50 cases, the corrected p-values were not significant compared to the significant uncorrected p-values.

	Species interaction	Estimate	Std. Error	P-value	Corrected P-value
TOTAL	cattle.cattle	0.155432	0.033367	3.19e-06 ***	1.60e-04 ***
	cattle.deer	-0.097167	0.022594	1.70e-05 ***	8.50e-04 ***
	cattle.horse	0.009801	0.022835	0.66776	1.00
	deer.deer	-0.366621	0.034057	< 2e-16 ***	1.00e-14 ***
	horse.deer	-0.324847	0.023407	< 2e-16 ***	1.00e-14 ***
	cattle.geese	-0.10817	0.023427	3.89e-06 ***	1.95e-04 ***
	deer.geese	-0.301708	0.023017	< 2e-16 ***	1.00e-14 ***
	geese.geese	0.062997	0.032814	0.05488 .	1.0
	horse.geese	0.058802	0.022442	0.00879 **	0.44
	horse.horse	0.006848	0.034447	0.84242	1.00
DEER	cattle.cattle	-0.07586	0.03857	0.04919 *	1.00
	cattle.deer	-0.21077	0.02481	< 2e-16 ***	1.00e-14 ***
	cattle.horse	-0.06495	0.02368	0.00609 **	0.305
	deer.deer	-0.40802	-0.03505	< 2e-16 ***	1.00e-14 ***
	horse.deer	-0.3112	-0.02278	< 2e-16 ***	1.00e-14 ***
	cattle.geese	-0.10197	0.02411	2.35e-05 ***	1.18e-03 ***
	deer.geese	-0.29609	-0.02237	< 2e-16 ***	1.00e-14 ***
	geese.geese	0.05749	0.03038	0.05846 .	1.00
	horse.geese	-0.06047	0.02057	0.00329 **	0.165
	horse.horse	-0.06302	0.03254	0.05277 .	1.00
CATTLE	cattle.cattle	-0.23395	0.03415	7.40e-12 ***	3.70e-10 ***
	cattle.deer	-0.01822	0.02281	0.424595	1.00
	cattle.horse	-0.07925	0.02317	0.000626 ***	3.13e-02 **
	deer.deer	0.17512	0.03356	1.80e-07 ***	9.00e-06 ***
	horse.deer	0.16318	0.02319	1.98e-12 ***	9.90e-11 ***
	cattle.geesee	0.14638	0.02381	7.86e-10 ***	3.93e-08 ***
	deer.geese	0.21437	0.02336	< 2e-16 ***	1.00e-14 ***
	geese.geese	0.01253	0.03366	0.709672	1.00

	horse.geese	-0.1178	0.02299	2.98e-07 ***	1.49e-05 ***
	horse.horse	-0.21098	0.03527	2.21e-09 ***	1.11e-07 ***
HORSE	cattle.cattle	0.2355	0.033316	1.56e-12 ***	7.80e-11 ***
	cattle.deer	0.002672	0.022531	0.90558	1.00
	cattle.horse	0.068476	0.022968	0.00287 **	0.144
	deer.deer	-0.208456	0.033668	5.96e-10 ***	2.98e-08 ***
	horse.deer	-0.196143	0.023388	< 2e-16 ***	1.00e-14 ***
	cattle.geese	-0.121995	0.023576	2.29e-07 ***	1.15e-05 ***
	deer.geese	-0.218824	0.023313	< 2e-16 ***	1.00e-14 ***
	geese.geese	0.012	0.034058	0.72459	1.00
	horse.geese	0.128369	0.023225	3.25e-08 ***	1.63e-06 ***
	horse.horse	0.148323	0.034835	2.06e-05 ***	1.03e-03 ***
GEESE	cattle.cattle	0.27447	0.04287	1.53e-10 ***	7.65e-09 ***
	cattle.deer	0.22154	0.02605	< 2e-16 ***	1.00e-14 ***
	cattle.horse	0.09947	0.02438	4.51e-05 ***	2.26e-03 **
	deer.deer	0.30318	0.03568	< 2e-16 ***	1.00e-14 **
	horse.deer	0.21436	0.02279	< 2e-16 ***	1.00e-14 ***
	cattle.geese	0.05753	0.02469	0.01982 *	0.991
	deer.geese	0.20399	0.02223	< 2e-16 ***	1.00e-14 ***
	geese.geese	-0.04279	0.03028	0.15763	1.00
	horse.geese	0.11224	0.02065	5.49e-08 ***	2.75e-06 ***
	horse.horse	0.09872	0.03341	0.00313 **	0.157

References

Begeleidingscommissie Beheer Oostvaardersplassen. (2018). *Advies Beheer Oostvaardersplassen. Report, Province of Flevoland.*

Clauss, M., Frey, R., Kiefer, B., Lechner-Doll, M., Loehlein, W., Polster, C., R�ssner, G. E., & Streich, W. J. (2003). The maximum attainable body size of herbivorous mammals: Morphophysiological constraints on foregut, and adaptations of hindgut fermenters. *Oecologia*, *136*(1), 14–27. https://doi.org/10.1007/s00442-003-1254-z

Cornelissen, P. (2017). *Large herbivores as a driving force of woodland-grassland cycles: The mutual interactions between the population dynamics of large herbivores and vegetation development in a eutrophic wetland* [Wageningen University]. https://doi.org/10.18174/396698

Coulson, T., Catchpole, E. A., Albon, S. D., Morgan, B. J. T., Pemberton, J. M., Clutton-Brock, T. H., Crawley, M. J., & Grenfell, B. T. (2001). Age, Sex, Density, Winter Weather, and Population Crashes in Soay Sheep. *Science*, *292*(5521), 1528–1531. https://doi.org/10.1126/science.292.5521.1528

Deagle, B. E., Thomas, A. C., McInnes, J. C., Clarke, L. J., Vesterinen, E. J., Clare, E. L., Kartzinel, T. R., & Eveson, J. P. (2019). Counting with DNA in metabarcoding studies: How should we convert sequence reads to dietary data? *Molecular Ecology*, *28*(2), 391–406. https://doi.org/10.1111/mec.14734

Demment, M. W., & Van Soest, P. J. (1985). A Nutritional Explanation for Body-Size Patterns of Ruminant and Nonruminant Herbivores. *The American Naturalist*, *125*(5), 641–672. https://doi.org/10.1086/284369

Hopcraft, J. G. C., Anderson, T. M., Pérez-Vila, S., Mayemba, E., & Olff, H. (2012). Body size and the division of niche space: Food and predation differentially shape the distribution of Serengeti grazers: Body size and the division of niche space. *Journal of Animal Ecology*, *81*(1), 201–213. https://doi.org/10.1111/j.1365-2656.2011.01885.x

Hopcraft, J. G. C., Olff, H., & Sinclair, A. R. E. (2010). Herbivores, resources and risks: Alternating regulation along primary environmental gradients in savannas. *Trends in Ecology & Evolution*, *25*(2), 119–128. https://doi.org/10.1016/j.tree.2009.08.001

Kartzinel, T. R., Chen, P. A., Coverdale, T. C., Erickson, D. L., Kress, W. J., Kuzmina, M. L., Rubenstein, D. I., Wang, W., & Pringle, R. M. (2015). DNA metabarcoding illuminates dietary niche partitioning by African large herbivores. *Proceedings of the National Academy of Sciences*, *112*(26), 8019–8024. https://doi.org/10.1073/pnas.1503283112

Mooij, J.H. (1992). *Behaviour and energy budget of wintering geese in the Lower Rhine area of north Rhine-Westphalia, Germany.*

Ripple, W. J., & Beschta, R. L. (2004). Wolves and the Ecology of Fear: Can Predation Risk Structure Ecosystems? *BioScience*, *54*(8), 755. https://doi.org/10.1641/0006-3568(2004)054[0755:WATEOF]2.0.CO;2

Sinclair, A. R. E., Mduma, S., & Brashares, J. S. (2003). Patterns of predation in a diverse predator–prey system. *Nature*, *425*(6955), 288–290. https://doi.org/10.1038/nature01934

Smit, C., Ruifrok, J. L., van Klink, R., & Olff, H. (2015). Rewilding with large herbivores: The importance of grazing refuges for sapling establishment and wood-pasture formation. *Biological Conservation*, *182*, 134–142. https://doi.org/10.1016/j.biocon.2014.11.047

Stapleton, T. E., Weinstein, S. B., Greenhalgh, R., & Dearing, M. D. (2022). Successes and limitations of quantitative diet metabarcoding in a small, herbivorous mammal. *Molecular Ecology Resources*, *22*(7), 2573–2586. https://doi.org/10.1111/1755-0998.13643

Thoresen, J., Vermeire, M.-L., Venter, Z., Wolfaard, G., Krumins, J. A., Cramer, M., & Hawkins, H.-J. (2021). Fire and herbivory shape soil arthropod communities through habitat heterogeneity and nutrient cycling in savannas. *Global Ecology and Conservation*, *25*, e01413. https://doi.org/10.1016/j.gecco.2020.e01413
